# Eco-Friendly Synthesis of Copper Oxide Nanoparticles Using Geranium *Pelargonium* x *hortorum* Leaf Extract and Its Biological Applications

**DOI:** 10.3390/pharmaceutics17121562

**Published:** 2025-12-04

**Authors:** Alexis Hernández-Guadarrama, Christian Andrea López-Ayuso, Raquel Garza-Hernández, Sarahi García-Carvajal, Ma. Concepción Arenas-Arrocena, A. Berenice Aguilar-Guadarrama, Laura Susana Acosta-Torres

**Affiliations:** 1 Escuela Nacional de Estudios Superiores, Unidad León, Universidad Nacional Autónoma de México, León 37684, Mexico; alopeza@enes.unam.mx (C.A.L.-A.); scarvajalg@enes.unam.mx (S.G.-C.); carenas@enes.unam.mx (M.C.A.-A.); 2 Interdisciplinary Research Laboratory (LII), Nanostructures and Biomaterials Area, León 37684, Mexico; 3 Centro de Investigaciones en Óptica, León 37150, Mexico; rgarza@cio.mx; 4 Centro de Investigaciones Químicas-IICBA, Universidad Autónoma del Estado de Morelos, Cuernavaca 62209, Mexico; baguilar@uaem.mx

**Keywords:** *Geraniaceae*, *P. hortorum*, biological activity, green synthesis, antimicrobial, antifungal

## Abstract

**Background/Objectives**: The main objective of this study is to report the green synthesis of copper oxide nanoparticles (CuONPs) using an aqueous extract from *Pelargonium* x *hortorum* (*P. hortorum*) leaves. It also aims to evaluate its biological activity as well as assess its cytotoxic effects on human gingival fibroblasts (HGFs). **Methods**: Copper oxide nanoparticles (CuONPs) were synthesized through chemical precipitation using an aqueous extract from *P. hortorum* leaves. These CuONPs were characterized with various techniques, including UV–Vis, Fourier transform infrared (FT-IR), X-ray diffraction (XRD), X-ray photoelectron spectroscopy (XPS), and transmission electron microscopy (TEM). **Results**: The UV–Vis spectrum showed a characteristic absorption peak for CuONPs. FT-IR spectroscopy identified alkoxide and aromatic groups associated with flavonoids and phenolic compounds from *P. hortorum*. The Cu–O bond was also observed in the same analysis. XRD confirmed that the CuONPs had a monoclinic CuO structure and XPS revealed copper was in the Cu (II) oxidation state bonded to oxygen, consistent with CuO. The nanoparticles were spherical with an average size of 40–53 nm as shown by TEM. The biological activities of CuONPs were tested against *Streptococcus mutans* (*S. mutans*) and *Candida albicans* (*C. albicans*). The minimum inhibitory concentration (MIC) was 20 µg/mL. Cytotoxicity tests on human gingival fibroblasts (HGFs) after 24 h showed a non-linear, dose-dependent cell viability profile, indicating CuONPs did not exhibit cytotoxicity within the tested range and could even promote cell proliferation at low and intermediate concentrations. **Conclusions**: This study successfully synthesized CuONPs via a green method, highlighting its potential as a biocompatible antimicrobial and antifungal agent.

## 1. Introduction

Nanomaterials have attracted interest due to their physicochemical properties promoted by their morphology and size (1–100 nm). They can be applied to a wide range of purposes such as therapeutic infections; anticancer, antitumor, and antimicrobial treatments; drug design; anti-inflammatory activity; oral diseases; and antioxidant properties [1,2,3]. Due to different biological activities, several methods have been employed for the development of nanomaterials, including sol–gel, chemical precipitation, hydrothermal, reverse micelle, and sonochemical methods [4,5,6]. Specifically, the chemical precipitation method offers cheap chemicals and mild reaction conditions, it is easy to scale-up, it does not require reducing agents, and it has a customizable treatment process design. Furthermore, when this method is combined with the extraction of natural products, green synthesis is developed, based on the elimination of hazardous substances in the manufacturing process of chemical products [7,8,9].

An extensive variety of nanomaterials using green synthesis have been explored, for example, ZnO [10], TiO_2_ [11], MnO_2_ [12], CeO_2_ [13], FeO [14], and CuO [15]. Particularly, CuONPs are nontoxic materials that exhibit good antimicrobial and antifungal properties [16,17]. The preparation of CuONPs by means of green synthesis using plant extracts has been demonstrated to be sustainable, eco-friendly, and cost-effective; consequently, the use of toxic chemicals is reduced, and the environmental impact is minimal [18].

**Figure 1:**
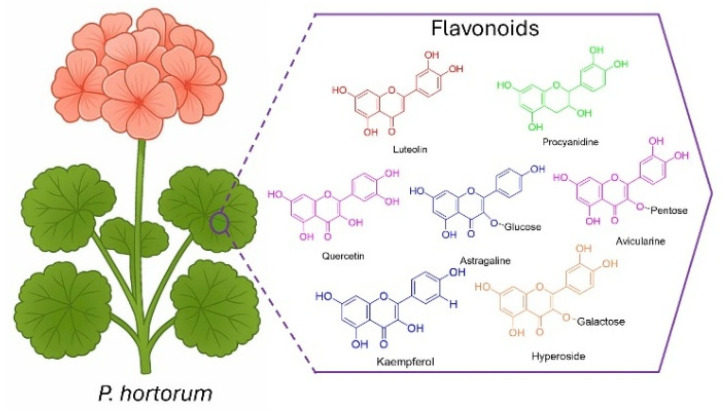
*Geraniaceae* plant (*P. hortorum*) and compounds identified.

Conversely, the *Geraniaceae* family consists of over 840 species, classified into six genera: *Geranium*, *Pelargonium*, *California*, *Monsonia*, *Hypseocharis*, and *Erodium*. Many species from this family have been traditionally used for medicinal purposes, particularly in the treatment of upper respiratory tract infections, such as bronchitis, the common cold, and sinusitis [19]. The therapeutic properties of these plants are primarily attributed to their diverse and rich phytochemical composition. In our research group, UPLC analysis confirmed the presence of several secondary metabolites in the aqueous leaf extract, including flavonoids, tannins, sesquiterpenes, phenolic acids, cinnamic acids, coumarins, monoterpenes, and polyphenols [20], as shown in Figure 1.

*P. hortorum*, *P. graveolens*, *P. reniforme*, *P. sidoides*, and *P. radula* are included among the most important *Pelargonium* species [21,22]. They have gained attention for their medicinal applications and their role in nanotechnology. Recent studies have demonstrated the potential of *Pelargonium* extracts in the green synthesis of various nanoparticles, such as ZnONPs [23], AgNPs [24], CeO_2_NPs [25], and AuNPs [26], as shown in Table 1. The biosynthesis of these nanoparticles using plant extracts presents an eco-friendly alternative to conventional chemical methods, reducing toxicity and their environmental impact while enhancing their biomedical and industrial applications. The *Pelargonium* species continues to be a promising source for both traditional medicine and modern pharmaceutical and technological advancements.

Until now, there were no studies or scientific reports documenting the synthesis of copper oxide nanoparticles (CuONPs) using *Pelargonium hortorum* leaf extract. In this sense, this work focuses on the synthesis, characterization, and potential applications of CuONPs derived from *P. hortorum* extracts.

## 2. Materials and Methods

### 2.1. Materials and Plant Sources

All reagents were purchased and used without purification. Copper chloride II CuCl_2_, Sigma-Aldrich (St. Louis, MO, USA, purity > 99.9%) and deionized water (DI water) were used as copper precursors and solvents, respectively. Fresh leaves of *P. hortorum* L.H. Bayley (*Geraniaceae*) were obtained in León, Mexico (21°02′41.6 N 101°40′13.2 W), at the beginning of December. A specimen was taxonomically identified by Dr. Sol Cristians Niizawa and deposited at the Faculty of Science Herbarium-UNAM, FCME, with voucher No. 182314 [20].

### 2.2. Green Synthesis of Copper Oxide Nanoparticles (CuONPs)

The leaf extract was obtained by the maceration of approximately 6.07 g of fresh leaves, which were rinsed and dried at room temperature. Then, leaves were ground in a mortar and added to a flask with 50 mL of DI water. The solution was maintained under boiling conditions for 15 min at a constant temperature of 89–92 °C. The solution was then filtered using Whatman filter paper (4–12 mm) to obtain an aqueous *P. hortorum* extract.

Nanoparticles were synthesized using 40 mL of 0.1 M CuCl_2_ as a copper precursor, and 10 mL of the aqueous leaf extract of *P. hortorum* was added. Then the solution was mixed and stirred at 500 rpm at 50 °C for 2 h. The change in color from green to brown in the solution was considered a preliminary confirmation of CuONP formation. Additionally, the solution was stirred for 24 h at room temperature [28]. Finally, the synthesized CuONPs were thermally annealed at 600 °C until crystallization was observed [29].

### 2.3. Characterization

Optical absorbance spectra were measured with a Multiskan Go UV–VIS spectrophotometer (Thermo Fisher Scientific, Waltham, MA, USA) in a spectral range between 200 and 600 nm at a resolution of 1 nm. The identification of functional groups was carried out in a Nicolet 6700 FT-IR mode ATR Fourier transform infrared spectrometer from Thermo Fisher Scientific™ ranging between 480 cm^−1^ to 4000 cm^−1^. The crystalline structure of the CuONP powder was determined by X-ray diffraction (XRD) using a D2 Phaser Bruker (Billerica, MA, USA) diffractometer with CuKα radiation at a wavelength of 1.5406 Å. The scan 2θ range was from 20° to 80° with a step of 0.022° and speed time of 2.64°/min. The chemical compositions and oxidation states of the CuONP powder were determined by X-ray photoelectron spectroscopy (XPS). The XPS analysis was carried out in the spectrometer K-Alpha (Thermo Scientifi, Waltham, MA, USA) using monochromatic Al-Kα radiation (1486.7 eV) as an excitation source and a hemispherical analyzer with an energy resolution of 0.25 eV. Charging corrections in the binding energy were applied by aligning the energy of the C1s peak at 284.8 eV. The morphology and elemental analysis were obtained with a JSM-7800F (JEOL, Akishima, Tokyo, Japan) field emission scanning electron microscope equipped with an energy dispersive X-ray (EDX) detector from Oxford Instruments. Surface morphology of the synthesized products was examined using transmission electron microscopy (TEM) on a JEM-1010 (JEOL, Akishima, Tokyo, Japan) microscope.

### 2.4. Antimicrobial Test

Antimicrobial tests were performed using *Streptococcus mutans* (ATCC^®^ 25175™) and *Candida albicans* (ATCC^®^ 90028™), obtained from the American Type Culture Collection (ATCC, Manassas, VA, USA). These strains were selected due to their clinical relevance in oral infectious diseases, with *S. mutans* being a primary etiological agent of dental caries and *C. albicans* a predominant fungal pathogen associated with oral candidiasis. The *S. mutans* cell line (strain designation NCTC 10449 [IFO 13955]) was obtained from the American Type Culture Collection (ATCC, Manassas, VA, USA); it is a whole-genome sequenced bacterial type strain isolated from carious dentine and is classified as Biosafety Level 1 (BSL-1) according to the ATCC (*Streptococcus mutans* Clarke-25175|ATCC). The *C. albicans* cell line (strain designation NCCLS 11) was also obtained from the American Type Culture Collection (ATCC, Manassas, VA, USA); it is a non-type strain, also classified as BSL-1 (*Candida albicans* (Robin) Berkhout 90028|ATCC). A young culture was prepared by inoculating a bacterial strain onto Mueller–Hinton agar (BD Bioxon, Mexico City, Mexico) and incubating it for 24 h prior to the experiment. Colonies of bacteria with similar morphology and size were placed in a 0.85% sodium chloride solution and the turbidity was measured using a densitometer (Grant Instruments™ DEN-1B, Cambridgeshire, UK) until the solution reached a turbidity of 0.5 McFarland scale. This opacity equated to 1 × 10^8^ CFU/mL^−1^.

### 2.5. Disk Diffusion and Microdilution Method

The CuONPs were analyzed at a concentration of 471 µg/mL; to achieve this, 1 mL of the synthesis solution was placed in an Eppendorf tube and centrifuged at 13,000 rpm for 30 min. The resulting supernatant was then removed, and 0.02 mL of sterile distilled water was added to the pellet. Subsequently, the pellet was impregnated with the experimental groups and placed on an agar plate (Mueller–Hinton for *S. mutans* and Sabouraud dextrose agar for *C. albicans*, Sigma-Aldrich^®,^ (St. Louis, MO, USA); 2% chlorhexidine (FGM, Mexico City, Mexico) and sterile water were used as controls. Thereupon, the plates were incubated at 37 °C for 24 h. The inhibition zone diameters were determined by the inhibition zone of the experimental groups. Furthermore, the zone of inhibition (ZOI) diameters were measured in millimeters (mm). The microdilution method was used to evaluate the growth of *S. mutans* and *C. albicans* at 24 h in the presence of CuONPs to determinate the minimum inhibitory concentration (MIC). Finally, the interpretation was based on the guidelines published by the National Committee for Clinical Laboratory Standards (NCCLS-CLSI 2021) [30]. All tests were performed in triplicate from three independent experiments (*n* = 9).

### 2.6. Cytotoxicity Assay

The HGFs were obtained from a gingival tissue biopsy taken during third molar surgery on a 21-year-old patient who had previously signed an informed consent form. The protocol was approved by the internal bioethics committee at the Leon Campus of the ENES, with registration number CE_16/004_SN. Following the fourth cell division, the HGFs were sent to the Oral and Maxillofacial Pathology department at the ENES-Leon Campus for characterization, in line with previous reports by our study group [20]. The HGFs were then inoculated at a ratio of 1:3 in 96-well plates and incubated for 24 h in fresh culture medium for cell studies.

The cytotoxic effect of CuONPs was evaluated using HGFs in eighth cell division at a density of 2 × 10^5^ cells per mL, cultured in 96 microwell plates, and incubated with 0 to 471 µg/mL CuONPs for 24 h and maintained at 37 °C, 5% CO_2_, and 95% relative humidity. After exposure, the number of viable cells was determined by the standard 3-(4,5-dimethylthiazol-2-yl)-2,5-diphenyltetrazolium bromide (MTT) method by incubating CuONP-treated cells with 0.2 mg/mL MTT in fresh culture medium for 4 h. Formazan crystals were dissolved with 0.1 mL of dimethyl sulfoxide (DMSO, Karal, Guanajuato, Mexico) and the results were analyzed on a Multiskan GO™ at 570 nm. Experiments were performed in triplicate (*n* = 9) from two independent experiments and results were expressed as a percentage of viable cell numbers compared to the control (untreated) group.

## 3. Discussion of Results

### 3.1. UV–Visible Spectroscopic Analysis

**Figure 2:**
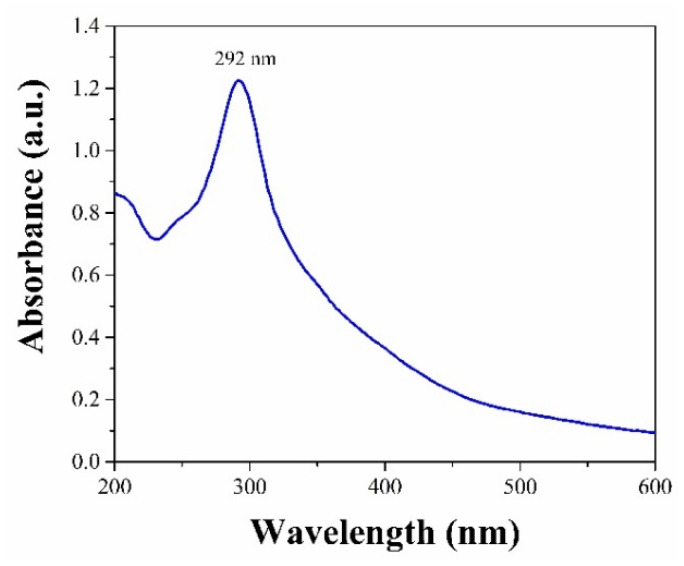
UV–Vis spectrum of CuONPs.

The CuONP absorbance spectrum shows a broad peak between 250 and 300 nm, which is related to the formation of CuONPs with the extract. The maximum absorption peak (λ_max_) at 292 nm is indicative of this kind of compound, associated with the presence of coumarins, tannins, and flavonoids [20,31,32], as shown in Figure 2.

### 3.2. Fourier Transform Infrared (FT-IR) Analysis

**Figure 3:**
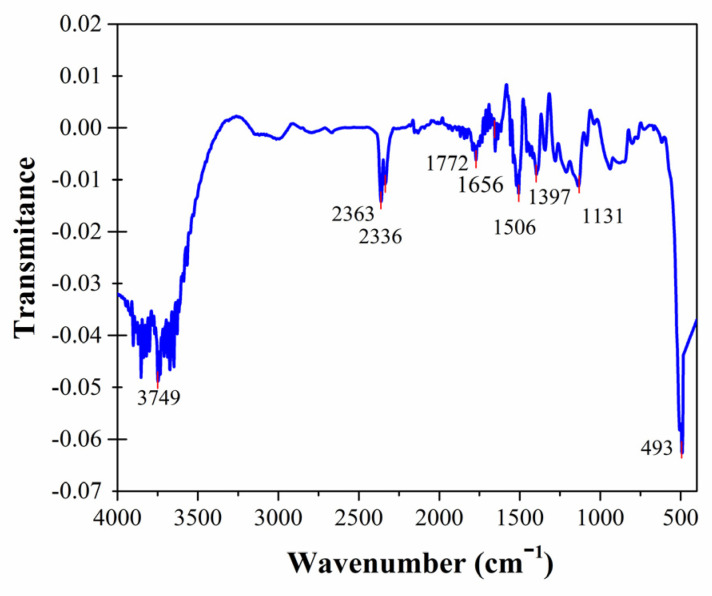
FT-IR spectrum of CuONPs.

The functional groups from CuONPs synthesized through *P. hortorum* extract were assigned through FT-IR spectra analysis. There were several strong peaks indicative of OH-stretching vibrations observed, mainly the greatest peak found at 3749 cm^−1^, with similar peaks at 3500 cm^−1^ in the FT-IR spectra of CuONPs reported previously [32]. The peak located at 1656 cm^−1^ was associated with the presence of functional group carbonyl [33], while alkoxide (O-R, stretching), aromatic (C=C, stretching), and alkane (C-H, bending) groups were assigned by the signals observed between 1400 cm^−1^ and 1100 cm^−1^ [34]. The signal at 493 cm^−1^ was related to the formation of a Cu-O bond coming from the CuONPs. Therefore, the metal–oxygen frequencies observed for CuONPs agree with those previously reported [35,36,37], as shown in Figure 3.

### 3.3. X-Ray Diffraction (XRD) Analysis

**Figure 4:**
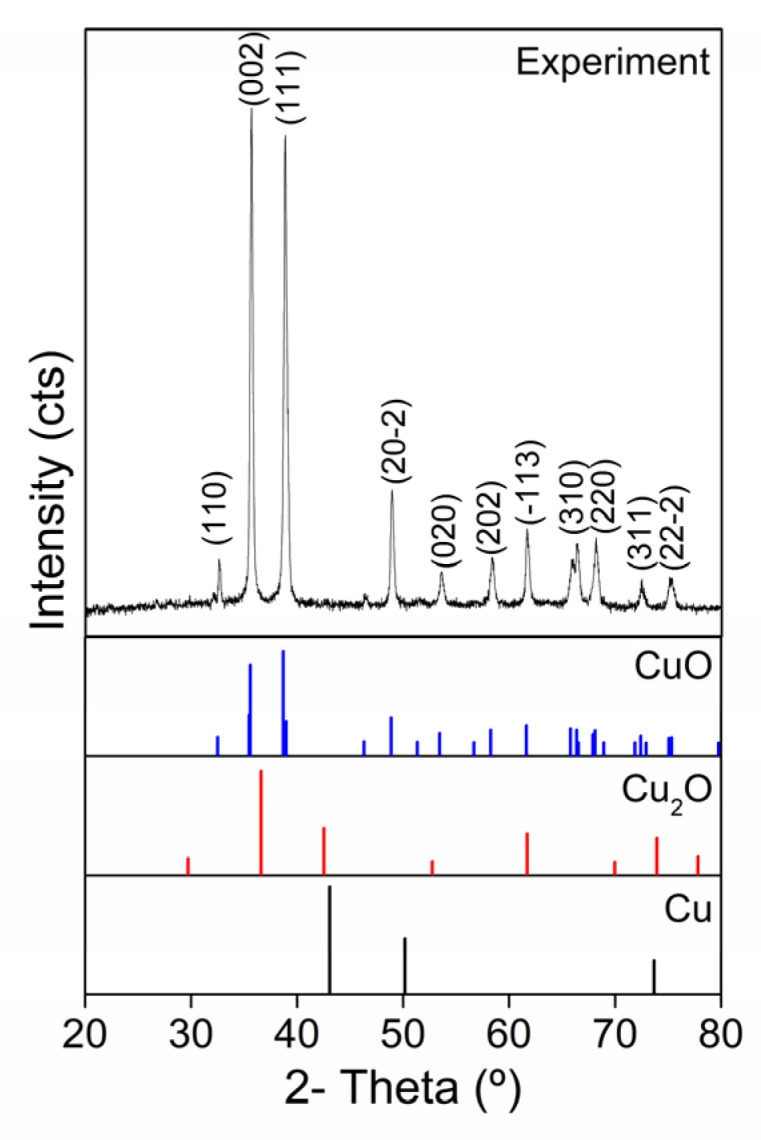
XRD analysis of CuONPs.

X-ray diffraction was used to determine the crystalline structure of CuONPs. A comparison of the experimental diffractogram and the Crystallography Open Database (COD) patterns of three structures, cubic-Cu (96-151-2505), cubic-Cu_2_O (96-100-0064), and monoclinic CuO (96-901-5925), is shown. The discernment between monoclinic and cubic structures was based on the position of the most intense experimental diffraction peak, which is located at 35.6° and corresponds to the [002] plane of the monoclinic CuO phase. Additionally, the experimental diffraction peaks observed at 32.6°, 35.6°, 38.8°, 48.9°, 53.7°, 58.3°, 61.7°, 66.3°, 68.3°, 72.6°, and 75.2° are attributed to the [110], [002], [111], [202], [020], [202], [113], [310], [220], [311], and [222] planes, which are in accordance with those observed in the CuO pattern. A complete oxidation of copper is observed due to the lack of detection of another peak for the Cu_2_O or Cu phases [38,39,40,41]. The crystallite size was obtained by applying the Scherrer equation, considering the width and position of the peak of the plane [002]. The crystallite size calculated was 33.8 nm, as shown in Figure 4.

### 3.4. Energy Dispersive X-Ray (EDX) Analysis

**Figure 5:**
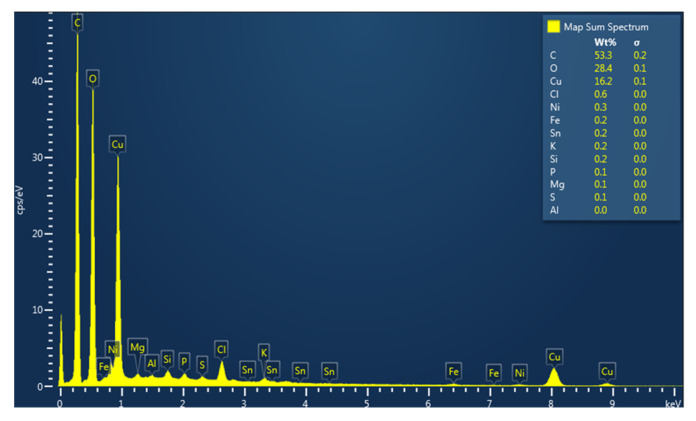
EDS spectrum for CuO nanoparticles (composition is in weight concentration).

The semiquantitative composition of CuONPs was obtained through energy dispersive X-ray (EDX), resulting in nanoparticles mainly comprising O (oxygen, 28.4%), C (carbon, 53.3%), and Cu (copper, 16.2%), as shown in Figure 5. Traces of other elements such as Cl (Chlorine, 0.6%), Ni (Nickel, 0.3%), and Fe (Iron, 0.2%) could have originated from the natural extract [32]. The outcome is in accordance with the findings of Peternela et al., who reported the EDX analysis of green synthesized CuO nanoparticles [42].

### 3.5. X-Ray Photoelectron Spectroscopy (XPS) Analysis

**Figure 6:**
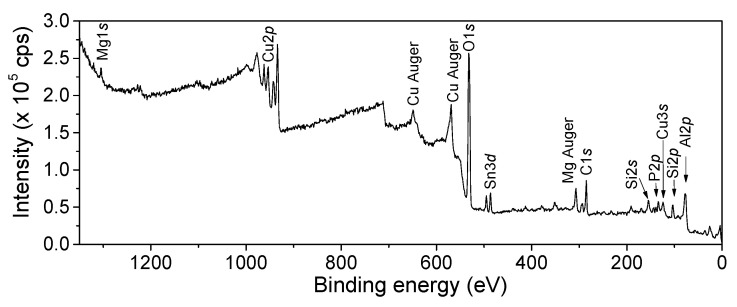
Survey spectrum of CuO nanoparticle powder obtained by XPS.

Figure 6 shows the survey spectrum for the CuONPs. In the spectrum, it is possible to see other highly sensitive orbitals and Auger peaks related to copper. Additionally, some impurities were detected, for example, carbon, aluminum, magnesium, tin, and silicon. Carbon is an element that is expected to be found, due to the fact that carbon can be easily absorbed from the atmosphere. On the other hand, silicon and other metals could have come from the extract of the plant.

**Figure 7:**
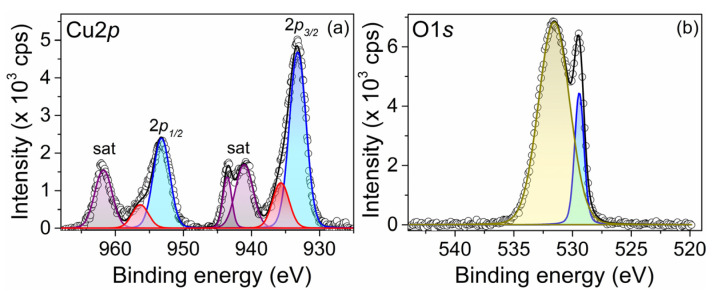
XPS spectra (**a**) Cu2p and (**b**) O1s core levels for CuONPs. The colors are intended to differentiate each of the species observed in the spectrum.

High-resolution spectra of Cu2p and O1s core levels were fitted using AAanalyzer software (version 2.04). The Cu2p region is constituted by Cu 2p_3/2_ and Cu 2p_1/2_ core levels. A high-intensity doublet peak was observed at 933.2 ± 0.10 eV with a spin–orbit splitting of 20.0 eV associated with CuO (blue peaks). The satellite peaks centered at 941.0, 943.5, and 961.7 eV are features of a CuO compound (purple peaks). A second doublet located at 935.7 eV with a spin–orbit separation of 20.6 eV is related to CuCl_2_ (red peaks), coming from the copper precursor used in the synthesis (Figure 7a). Alternatively, the O1s spectrum is composed of two peaks, with one located at 529.4 eV and 531.5 eV corresponding to O-Cu and O-C, respectively [43,44,45,46] (Figure 7b). Cu2p and O1s line intensities, corrected for the appropriate atomic sensitivity factors (ASFs), were used to determine the surface atomic composition of the CuO sample. ASF values were obtained from the Thermo database table. Atomic concentration percentages obtained through XPS for Cu (II) and O were 35% and 65%, respectively. The results obtained through XPS confirm the previous results obtained through XRD.

### 3.6. Transmission Electron Microscopy (TEM) Analysis

**Figure 8:**
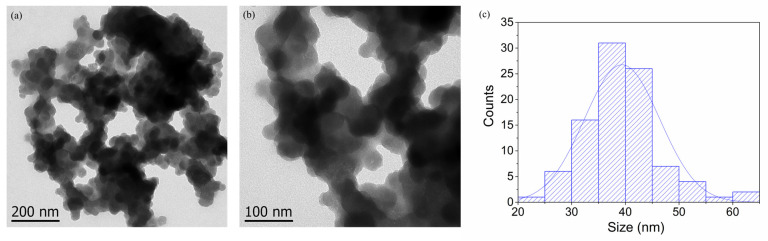
Transmission electron microscopy (TEM) images at (**a**) 200 nm and (**b**) 100 nm of CuONPs, and (**c**) size distribution histogram.

Transmission electron microscopy (TEM) is the most widely used technique for determining the morphological features and sizes of nanostructures. TEM micrographs of CuONPs demonstrated that the nanoparticles exhibit a spherical shape with an average particle size of 39.3 ± 7.01 nm, as shown in Figure 8.

### 3.7. Antimicrobial Activity

**Figure 9:**
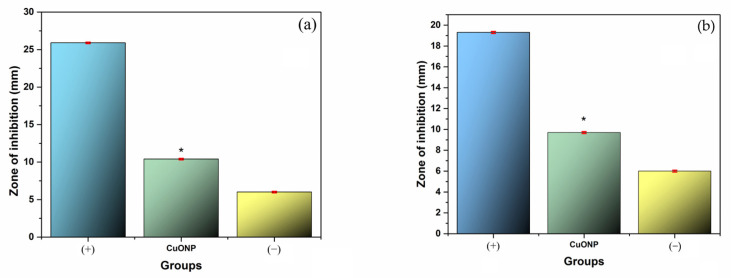
Antimicrobial effect of CuONPs evaluated by agar diffusion against (**a**) *S. mutans* at 24 h, positive control: chlorhexidine (+); (**b**) *C. albicans* at 24 h, positive control: amphotericin (+), negative control: sterile water (−). Each value in the graph represents the mean and SD. One-way ANOVA and Tukey’s post hoc test were performed. * Represents a significant difference (*p* < 0.05), *n* = 9 in triplicate experiments.

**Figure 10:**
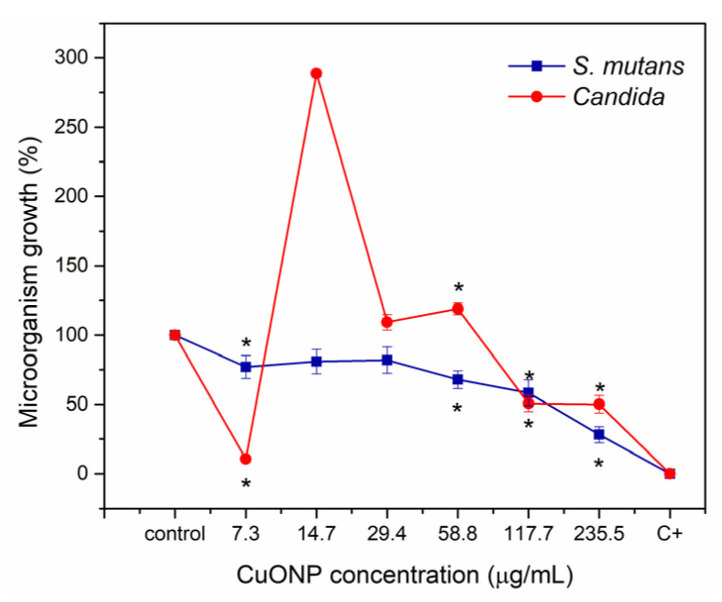
Antimicrobial effect of CuONPs by microdilution method to evaluate the growth percentage of *S. mutans* at 24 h and identify MIC at 235.5 µg/mL^−1^ and *C. albicans* at 117.7 µg/mL^−1^. Each value on the graph represents the mean and S.D. One-way ANOVA and Tukey post hoc were performed, * representing the concentrations with a significant difference. *p* < 0.05, *n* = 9 in duplicate experiments.

The antimicrobial potency of CuONPs was examined using the agar diffusion tests for assessing the antibacterial activity and fungal activity of CuONPs, measuring the diameter of the zone of inhibition (ZOI) of nanoparticles against *S. mutans* ATCC 25175 bacteria and *C. albicans* ATCC 90028 fungi. The ZOIs are summarized in Figure 9. The MIC was determined to be 235.5 µg/mL for *S. mutans* and 117.7 µg/mL for *C. albicans*, as shown in Figure 10.

CuONPs showed ZOIs against both *S. mutans* and *C. albicans*, which suggests that they have moderate but measurable antimicrobial activity against Gram-positive bacteria (*S. mutans*) and yeast-like fungi (*C. albicans*) (*p* > 0.05).

This finding is consistent with studies reported in the literature that have demonstrated the ability of CuONPs to generate reactive oxygen species (ROS), alter membrane permeability, and destabilize intracellular components of pathogenic microorganisms [47,48]. Additionally, the inhibition zones generated by CuONPs were smaller than those observed with positive control; it is important to highlight that the inhibitory effect was maintained in both cases, which underlines their potential as an alternative or adjuvant antimicrobial agent.

The use of CuONPs represents an interesting alternative due to their antimicrobial properties, their possibility of incorporation into biomaterials or biomedical devices, and their potential synergistic effect with other compounds [47]. However, the limited size of the inhibition zone identified suggests that higher concentrations or improved formulations are required to achieve effectiveness comparable to current clinical standards [49].

### 3.8. Cytotoxicity

**Figure 11:**
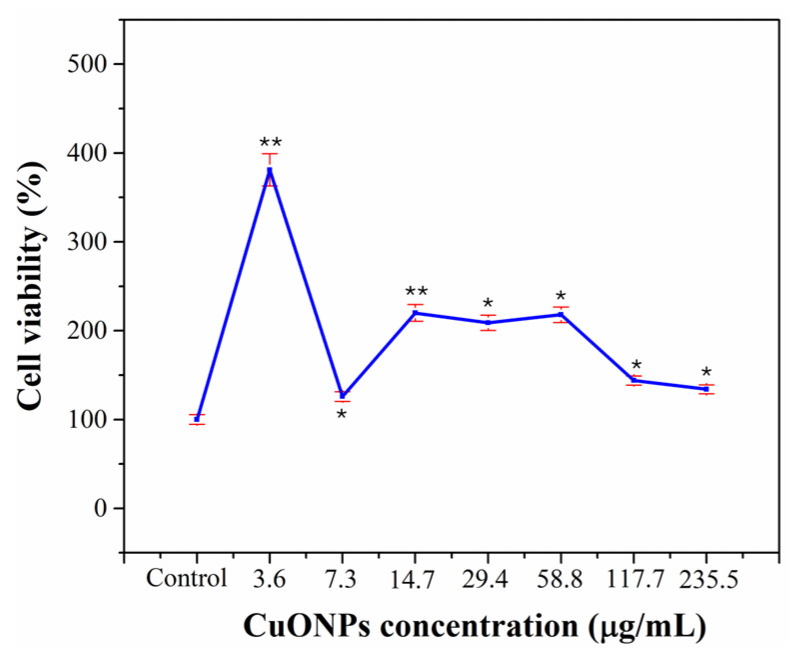
Cytotoxicity was determined by colorimetric MTT bioassay with HGFs at different concentrations (0–235.5 µg/mL) exposed for 24 h. Each value in the figure represents the percentage of the mean and SD. One-way ANOVA and Tukey’s post hoc test were performed; * represents concentrations with a significant difference (* *p* ≤ 0.001, ** *p* ≤ 0.05), *n* = 9 in duplicate experiments.

Evaluation of the cytotoxicity of CuONPs on HGFs after 24 h exposure did not reveal any cytotoxic effects. At the lowest concentration tested (3.6 µg/mL), a notable increase in cell viability was observed (~381% compared to the control), which could suggest a proliferative effect. At intermediate concentrations (14.7–58.8 µg/mL), viability remained high (~218–220%), with a slight reduction compared to the observed peak. At higher concentrations (117.7 and 235.5 µg/mL), a decrease in viability was observed (~144% and ~134%, respectively), but in all cases it remained above 100% (Figure 11), indicating a biocompatibility profile.

This proliferative effect has been previously reported in other cell types, where the controlled release of Cu (II) ions can modulate cell signaling pathways involved in cellular proliferation, differentiation, and antioxidant response in certain contexts [50,51]. In the context of tissue regeneration, in connective tissues such as periodontal or gingival, it could enhance bioactive agents in scaffolds or dental materials with regenerative properties [50]. However, proliferative or hormesis-like effects have been described for several metallic nanoparticles, with low concentrations stimulating and high concentrations inhibiting cellular functions [52,53]. Our results indicated that CuONPs are likely to stimulate metalloproteinase activity in fibroblasts, inducing the activation of intracellular signaling pathways that regulate various cellular processes such as proliferation, survival, migration, and extracellular matrix production [52,53,54]. Our results have demonstrated the potential of CuONPs as bioactive agents with proliferative properties in gingival tissue cells; however, further studies are suggested to elucidate the underlying molecular mechanisms and to validate their in vivo behavior.

## 4. Conclusions

In this study, copper oxide nanoparticles (CuONPs) were successfully synthesized using *P. hortorum* leaf extract, demonstrating the suitability of plant-mediated green synthesis. Its characterization was confirmed for its crystalline structure, morphology, and chemical composition through various analytical techniques (UV–Vis, FT-IR, XRD, EDX, XPS, and TEM). Furthermore, this study demonstrated that CuONPs exhibit antimicrobial and antifungal activity, effectively inhibiting the growth of *S. mutans* and *C. albicans*, two clinically relevant pathogens associated with oral infections. Furthermore, cytotoxicity analysis revealed no evidence of cytotoxic effects across the evaluated concentration range. Therefore, these results support their potential to serve as promising candidates for biomedical applications.

## Figures and Tables

**Table 1:** Reported nanoparticles synthesized using *Pelargonium* species.

Pelargonium Species	Nanoparticle Type	Main Applications
*P. hortorum*	ZnONPs	Antibacterial, photocatalytic [23]
*P. graveolens*	AgNPs	Antimicrobial, antioxidant [24]
*P. reniforme*	AgNPs	Antifungal, antibacterial [25]
*P. sidoides*	CeO_2_NPs	Antimicrobial, antioxidant, [26]
*P. graveolens*	AuNPs	Catalysis, biomedical [27]

## Data Availability

The raw data presented in this work is available on request.
